# Healthy aging and geroscience: aligning European public expectations with prevention-first policy

**DOI:** 10.1016/j.jnha.2026.100944

**Published:** 2026-08-18

**Authors:** Antoine Piau, Bruno Vellas

**Affiliations:** a Maintain Aging Research Team, Centre d'Epidémiologie et de Recherche en Santé des POPulations, Université de Toulouse, Inserm, Université Paul Sabatier, Toulouse, France; b Medical, Ethics and Innovation Department, Clariane Group, 75008 Paris, France; c I.H.U. HealthAge, Toulouse University Hospital, WHO Collaborating Center for Frailty, Clinical and Geroscience Research, and Geriatric Training, Toulouse, France

Population aging is a defining challenge for European societies, yet health systems and public discourse remain poorly attuned to the expectations of citizens across the continent. To our knowledge, few large-scale cross-national surveys have explored public perceptions of healthy aging and geroscience simultaneously. We therefore report findings from a recent European survey, conducted among 12,078 adults aged 18 years and older across six countries (France, Germany, Italy, Spain, Belgium, and the Netherlands), with samples representative by age, sex, socio-professional category, region of residence, and income [1].

The results reveal a strong and consistent consensus across all six countries. More than eight in ten respondents (84%) agreed that enabling the population to “age well” should be elevated to a major national priority, while only 32% believed their society was adequately prepared to face population aging. This concern is embedded in a wider sense of social relegation: 69% felt that older people are devalued within their society, and 40% considered that discussions about aging and old age remain a taboo topic [1]. These findings point to a widening gap between demographic reality and social readiness, which appears to be a shared European condition.

At the individual level, the survey reveals a familiar but nuanced paradox. Across the six countries, 38% reported feeling younger than their chronological age - a proportion rising to 46% in Spain - yet overall, 39% expressed anxiety about their own aging, with French, Italian, and Belgian respondents reporting the highest levels of concern. Among those who were worried, fears concentrated on disease onset (61%), loss of autonomy (60%), and cognitive decline (54%); lifestyle constraints and social isolation also featured prominently, underscoring that aging anxiety extends beyond the merely biomedical [1].

*These concerns point to a common structural gap in long-term care across Europe. Informal care* remains overwhelmingly dominant: 70% of respondents expected to rely primarily on family support in old age. By contrast, only 24% anticipated drawing on external structured support, whether public services or voluntary organizations, and 18% counted solely on themselves [1]. This imbalance - consistent across all six countries, though most pronounced in Southern Europe - underscores the limited perceived reliability of care systems, and the heavy personal and familial costs that current arrangements impose.

Encouragingly, support for prevention is both robust and consistent. Across the six countries, 81% were willing to undergo preventive health check-ups to anticipate age-related disease, 74% would make substantial lifestyle modifications, and 60% were open to using connected devices for continuous health monitoring [1]. Crucially, this disposition is not confined to any single country or age group; Spanish and Italian respondents were particularly willing to consider more innovative interventions, including biologically-oriented approaches to slowing aging, where efficacy was demonstrated. A majority (77%) also recognized that science can help people live better with the effects of aging, and 65% believed they could delay those effects [1].

These preferences align closely with the promise of geroscience. Aging is the dominant risk factor for most chronic diseases, and targeting its fundamental biological mechanisms offers the prospect of extending healthspan and compressing morbidity [[2], [3], [4]]. Yet awareness remains limited: 64% of European respondents had never heard of geroscience, with France and Belgium showing the lowest recognition rates (71% unaware). When the field was defined - as research and interventions aimed at understanding and improving the aging process - a large majority endorsed its value, mapping the concept spontaneously onto their stated priorities: remaining healthy for longer, preventing disease, reducing dependency, and containing public expenditure [5].

Taken together, these findings (Table 1) confirm a rare convergence across Europe: public concern about aging is high, willingness to invest in prevention is substantial, and latent support for geroscience exists wherever the field is adequately explained. The challenge is now institutional. Prevention must be treated as an entitlement, financed with the same seriousness as cure, delivered at scale through integrated community-based models supported by pragmatic digital tools, and designed to reduce the profound social inequalities in how aging is experienced. Healthspan must become a measurable policy objective across European health systems, not a rhetorical aspiration.

**Table 1:** Key findings — Healthy aging and geroscience: a European public perspective.

• In a cross-national European survey (n = 12,078; 6 countries; April - May 2026), 84% agreed that enabling everyone to ‘age well’ should be a national priority, but only 32% thought their society was prepared to face population aging [1]. • Readiness to act is high across all six countries (81% willing to undergo preventive check-ups, 74% willing to make major lifestyle changes), yet anxiety about aging is widespread (disease onset 61%, loss of autonomy 60%, cognitive decline 54%) [1]. • Long-term support remains overwhelmingly informal: 70% expect to rely primarily on family; only 24% anticipate drawing on external structured support (public services or associations) [1]. • Knowledge of geroscience was limited across Europe (64% had never heard of it, rising to 71% in France and Belgium), but after a brief definition respondents endorsed its objectives and mapped them onto their own aging priorities [1]. • A prevention-first architecture for healthy aging requires four shifts at European level: prevention as an entitlement, financed like care, delivered at scale through culturally adapted community models, and equitable by design.

Legend: OpinionWay for Clariane - European Aging Observatory (France, Germany, Italy, Spain, Belgium, Netherlands; n = 12,078; fieldwork April - May 2026) [1].

## Author contributions

A.P. conceptualized the article, interpreted the data, and wrote the original draft. B.V. contributed to data interpretation and critical revision of the manuscript. Both authors approved the final version.

## Declaration of Generative AI and AI-assisted technologies in the writing process

During the preparation of this work, the authors used Claude Sonnet 4.6 (Anthropic) for editing assistance and English language refinement only. The authors reviewed and edited all AI-assisted content and take full responsibility for the content of the publication.

## Funding

No funding was received for the preparation of this Letter. The survey cited was commissioned and funded by Clariane; no funding was provided to the authors for this manuscript.

## Declaration of competing interest

A.P. is employed by the Clariane Group, which commissioned the survey cited in this article. B.V. declares no competing interests.
